# Landscape structure influences natural pest suppression in a rice agroecosystem

**DOI:** 10.1038/s41598-023-41786-y

**Published:** 2023-09-21

**Authors:** M. P. Ali, Gemma Clemente-Orta, M. M. M. Kabir, S. S. Haque, M. Biswas, Douglas A. Landis

**Affiliations:** 1 Entomology Division, Bangladesh Rice Research Institute (BRRI), Gazipur, 1701 Bangladesh; 2 Department of Crop and Forest Sciences, AGROTECNIO Center, University of Lleida, Rovira Roure 191, 25198 Lleida, Spain; 3 Department of Geography, Presidency University, 86/1, College Street, Kolkata, West Bengal 700073 India; 4 Department of Entomology, Michigan State University, East Lansing, MI USA

**Keywords:** Agroecology, Biodiversity, Ecological modelling, Ecosystem services

## Abstract

Agricultural landscapes are constantly changing as farmers adopt new production practices and respond to changing environmental conditions. Some of these changes alter landscape structure with impacts on natural pest control, pesticide use, and conservation of biodiversity. In rice agroecosystems the effect of landscape structure on natural enemies and pest suppression is often poorly understood. Here we investigate the effect of landscape composition and configuration on a key pest of rice, the brown planthopper (*Nilaparvata lugens*). Using *N. lugens* as sentinel prey coupled with predator exclusions, we investigated landscape effects on herbivore suppression and rice grain yield at multiple spatial scales in two regions of Bangladesh. Ladybird beetles and spiders were the most abundant natural enemies of *N. lugens* with landscape effects observed at all scales on ladybird beetles. Specifically, ladybird beetles were positively influenced by road edges, and fallow land, while spiders were strongly influenced only by rice phenology. Predator exclusion cages showed that *N. lugens* abundance significantly increased in caged plots, reducing rice gain yield. We also used an estimated biocontrol service index that showed a significant positive relationship with landscape diversity and a significant negative impact on pest density and yield loss. These results suggest that promoting fallow lands and fragmented patches between rice fields could lead to more sustainable insect pest management in rice agroecosystems, potentially reducing the practice of prophylactic insecticide use.

## Introduction

Landscape structure is increasingly recognized as an important factor regulating biodiversity and ecosystem services in agricultural landscapes. Landscape structure is characterized by both landscape composition i.e. types and amount of habitats, and configuration i.e. shapes and spatial arrangement of elements, which interact to influence outcomes^1^. Heterogeneity is the number and proportions of different cover types and their complex spatial arrangement in the landscape. Increasing heterogeneity in landscape structure at local and landscape levels can increase the overall species pool, facilitate spillover of organisms across habitats, and increase the diversity and resilience of ecosystem functions^2^. Heterogeneity in agricultural landscapes is frequently critical to support the ecosystem services that come from biodiversity^3,4^. Specifically, biodiversity can strengthen the provision of important ecosystem functions like natural pest suppression (aka. biological control)^5,6^. Conversely, a lack of crop diversity caused by monocultures of a single crop at large scales can reduce pest suppression resulting in greater losses due to crop pests and diseases^5^.

Efforts to increase pest suppression in agroecosystems often utilize principles from conservation biological control to increase pest suppression^8–10^. Such approaches aim to increase the functional efficiency of natural enemy populations by managing habitats in or around crop to offer substitute sources of food, such as prey, pollen, and nectar, as well as providing refuge from agricultural disturbances^11,12^. More recently, these practices have been combined with insights from landscape ecology to inform the potential to design agricultural landscape for multiple ecosystem services^13^. Specifically, to design landscapes that support crop yield while enhancing other arthropod-based ecosystem services such as pollination, pest suppression, and decomposition^14^.

In tropical Asia, rice fields frequently have greater arthropod species diversity than other types of natural settings^15^ which can contribute to stable pest suppression. However, indiscriminate use of insecticides, combined with intensification of rice cultivation can decouple herbivore-environment interactions, resulting in frequent severe pest outbreaks^16^. Several studies have examined the impact of manipulating local landscape composition via habitat management to enhance biological pest control services in rice field^9,17,18^. For example, increasing compositional heterogeneity in rice landscapes enhances parasitoids^14^, while increasing local landscape diversity by adding nectar rich flowering plant near rice fields can increase the abundance of natural enemies and reduce crop damage^9,10^.

The tropical Asian rice landscape consists of a patchwork of landscape elements, including rice itself, other crops, fallow fields, and natural vegetation. The configuration of habitat patches surrounding rice paddies may have an impact on pests, natural enemies, and other elements of the agroecosystem by changing the range of host and prey resources or the ranges of microclimatic conditions^19,20^. Fragmentation of habitat can influence the abundance of insect pest and prey-predator dynamics in agroecosystems^19^. However, rice fields in Asia are generally connected by a vast network of bunds (narrow levees that allow irrigation and provide access to fields), which support semi-natural vegetation and can provide alternate food supplies or refuge to natural enemies^22^ and support dispersal through the rice agroecosystem. For example, predatory ladybird beetles (*Coccinella*), and egg parasitoids (*Anagrus* and *Oligosita* wasps) of some rice pests are generally found in wild grasses on rice bund^23^. In addition, vegetation in rice bunds provides habitat for spiders which disperse into establishing rice crops^15^. Both ladybird beetles and spiders are commonly observed in rice fields and can keep the key insect pest of rice, the brown planthopper (BPH, *Nilaparvata lugens* Stål, 1854) population below damaging levels. In addition, spiders are common and abundant predators that are recognized to be very effective predators of many rice insect pests worldwide^24,25^. *Pardosa pseudoannulata* is a most abundant spider species in rice fields and considered as an effective biocontrol agent of BPH in south Asia^26^. Ladybird beetles are also very effective predators in many places in the World.

Increasing studies indicate that manipulating compositional and configurational diversity of rice-associated habitats can improve the effectiveness of natural enemies in managing herbivores^9,10,18,24^. Most of these studies has been done at field and plot scales, with little attention to the larger landscape. Some studies showed that managing rice bunds improve resources for natural enemies (e.g., see differences between results from Yao et al.^17^ and Gurr et al.^9^ and those from Horgan et al.^18^, and Sann et al.^27^). In addition, Yao et al.^17^ use soybean and corn intercrops with rice which do not greatly enhance the ability of natural enemies to suppress planthoppers. However, Gurr et al.^9^ grew nectar-producing plants around rice fields, which enhanced predators and parasitoids significantly reduced populations of planthoppers. While local scale manipulations can be effective in increasing pest suppression, it remains to be determined how landscape structure influences their effectiveness. Success could be determined by the availability of adequate natural vegetation at previously unstudied scales, or by the configuration of rice production landscapes.

Recently, parts of Bangladesh are experiencing changes in agricultural landscapes due to climate change. Specifically, our study is focused on Patuakhali and Satkhira regions where salinity intrusion occurs every year due to high tidal water, and cyclones which surge saline water into the agricultural landscape. During the dry season in Patuakhali, farmers cannot grow rice due to a lack of fresh water for irrigation^28^. As a result, most rice production land currently remains fallow in Patuakhali during the Boro (winter) rice growing season concentrating natural enemies, especially ladybird beetles in the remaining rice. Moreover, in Bangladeshrice ecosystem, more than 375 beneficial arthropod species have been identified^29^ and several weeds and other ratoon crops in fallow land attract natural enemies by providing food and shelter (Ali, unpublished data). In this paper, we assess landscape structure embedded in two Bangladesh rice landscapes using various compositional and configurational metrics based on high-resolution satellite imagery. We then assessed the impact of landscape structure on two natural enemies of BPH in rice fields (ladybird beetles and spiders) and subsequent yield impacts in plots open or closed to these predators to disentangle the effects of landscape structure on natural pest control services in Bangladesh rice with implications for future landscape management. Specifically, we tested the following hypotheses:

1. Non-crop areas, i.e., fallow lands and natural habitats, have positive effects on NE abundance in rice agroecosystems.
2. Landscape with high diversity have positive effects on NE and negative effects on pest abundance and crop damage.

## Results

### Abundance of brown planthoppers and its natural enemies

**Figure 1:**
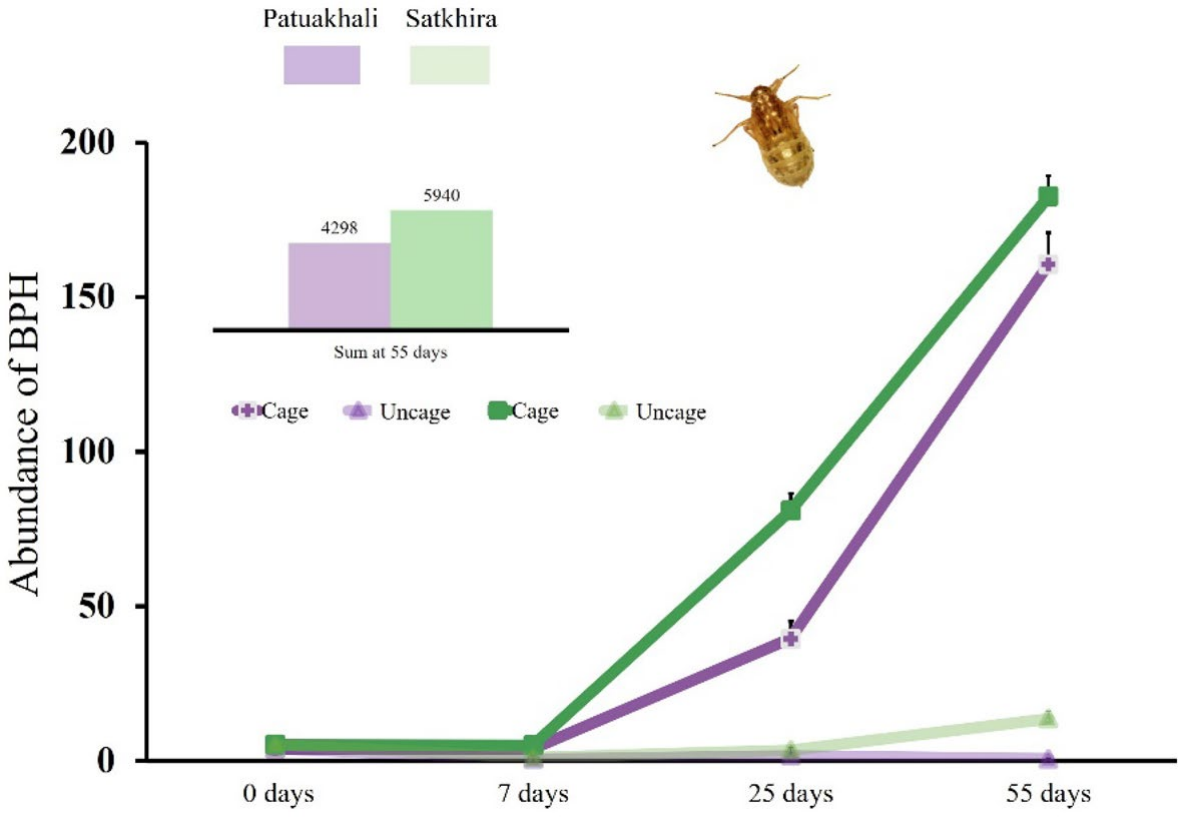
Abundance of brown planthopper (BPH) in each region and treatment at 0, 7, 25 and 55 days after release. Significant differences were found between two treatments, χ^2^ test (*p* < 0.05) in each region.

Brown planthopper (BPH) populations increased rapidly after seven days in both regions and were significantly higher in caged versus uncaged plots in both regions; Satkhira (χ^2^ = 189.25, *df* = 1, *p* < 0.001) and Patuakhali (χ^2^ = 140.31, *df* = 1, *p* < 0.001) (Fig. 1). BPH populations did not reach damaging levels outside of caged plots in the experimental fields (Fig. 1) while natural enemies were found in uncaged but not in caged plots. During the experiment we frequently observed natural enemies on the outside of the mesh net of cage plots (Fig. S1).

### Impact of natural pest control services on grain yield loss

**Figure 2:**
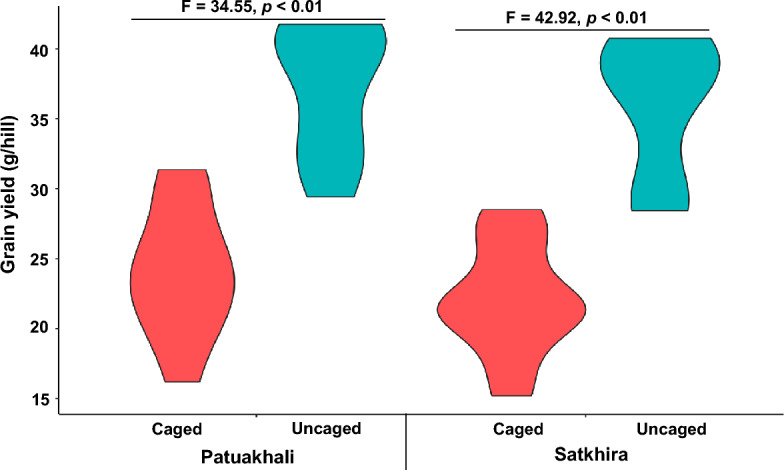
Impact of natural pest control services on grain yield in caged and uncaged plots with the mean and standard deviation measured in Patuakhali and Satkhira. The figure was prepared using R software.

The number of panicles per hill is the most important characteristic for rice grain yield^29^. Significant lower grain yield was recorded in caged versus uncaged plots in both regions (Fig. 2). The grain per hill was higher in uncaged plots both in Patuakhali (t = 5.87, *df* = 1, *p* < 0.001) and in Satkhira (t = 6.55, *df* = 1, *p* < 0.001). The average grain loss per hill ranged from 15.3 to 37.0% across the geographic locations. Grain yield also depended on year, with 15.3–34.7% grain loss in 2018–2019, and 21.5–37.0% in 2019–2020. Lower panicle numbers per hill were observed in caged versus uncaged plots, averaging 6.1–15.4% less panicles per hill in caged plots.

### Biological control services index

The estimated Biological Control Services Index (BSI) was 0.96 ± 0.07 in 2019 (range 0.90–0.98) and 0.97 ± 0.01(range 0.96–0.99) in 2020 at Patuakhali and 0.95 ± 0.03 (range 0.89–0.99) in 2019 and 0.78 (range 0.75–0.84) in 2020 at Satkhira, indicating highly effective biological control at each location in each year in unsprayed rice fields. Landscape structure did not show any significant impact on BSI across both years or spatial scales. But there was a significant positive correlation between BSI and landscape diversity index (SHDI) (Pearson's r = 0.47, *p* = 0.033) at 700 m, and (Pearson's r = 0.53, *p* = 0.015) at 200 m respectively. A significant negative correlation was found between BSI and pest density (Pearson's r = − 0.695, *p* = 0.0013) as well as a significant negative correlation between pest density and grain weight (Pearson's r = − 0.31, *p* < 0.001).

### Effects of rice landscape on natural enemies

Ladybird beetle abundance in rice fields responded to landscape structure at all four spatial scales studied (Table 1). The best model following AICc criteria was at 500 m scale and showed that the abundance of ladybird beetles was highly positively influenced by road and fallow land and, negatively by urban areas. Waterbodies had a positive effect on ladybird beetle abundance at 1000 m scale but in general the proportion of natural habitat or edge density was not significant in the models. Landscape effects were not significant for spiders which were negatively influenced by rice phenology in all models.

**Table 1:** The best effects model on ladybird beetles and spider abundance, as well as landscape variables selected using generalised linear mixed models in multimodel inference.

	1000 m				700 m				500 m				200 m			
	Variable	Est.	z	*p*	Variable	Est.	z	*p*	Variable	Est.	z	*p*	Variable	Est.	z	*p*
Ladybird beetles	(Intercept)	2.18	2.11	0.03	(Intercept)	2.14	1.777	0.07	(Intercept)	1.75	2.19	0.03	(Intercept)	1.87	1.98	0.04
	Water-bodies	0.21	2.65	0.008	Road	0.14	2.05	0.04	Fallow	0.38	2.4	0.01	Urban	0.16	2.84	0.003
									Road	0.12	2.07	0.04				
									Urban							
	AICc	324.6			AICc	327.62			AICc	324.9			AICc	325.41		
Spiders	(Intercept)	2.36	7.68	< 0.001	(Intercept)	2.36	7.68	< 0.001	(Intercept)	2.35	7.61	< 0.001	(Intercept)	2.35	7.9	< 0.001
	Phenology-mid	− 0.27	1.96	0.01	Phenology-mid	− 0.27	1.95	0.04	Phenology-mid	− 0.27	0.04		Phenology-mid	− 0.27	1.95	0.04

Models were fitted at four spatial scales surrounding the sampled fields, ranging from 200 to 1000 m. Only significant variables in the best models (ΔAICc < 2) are presented.

**Figure 3:**
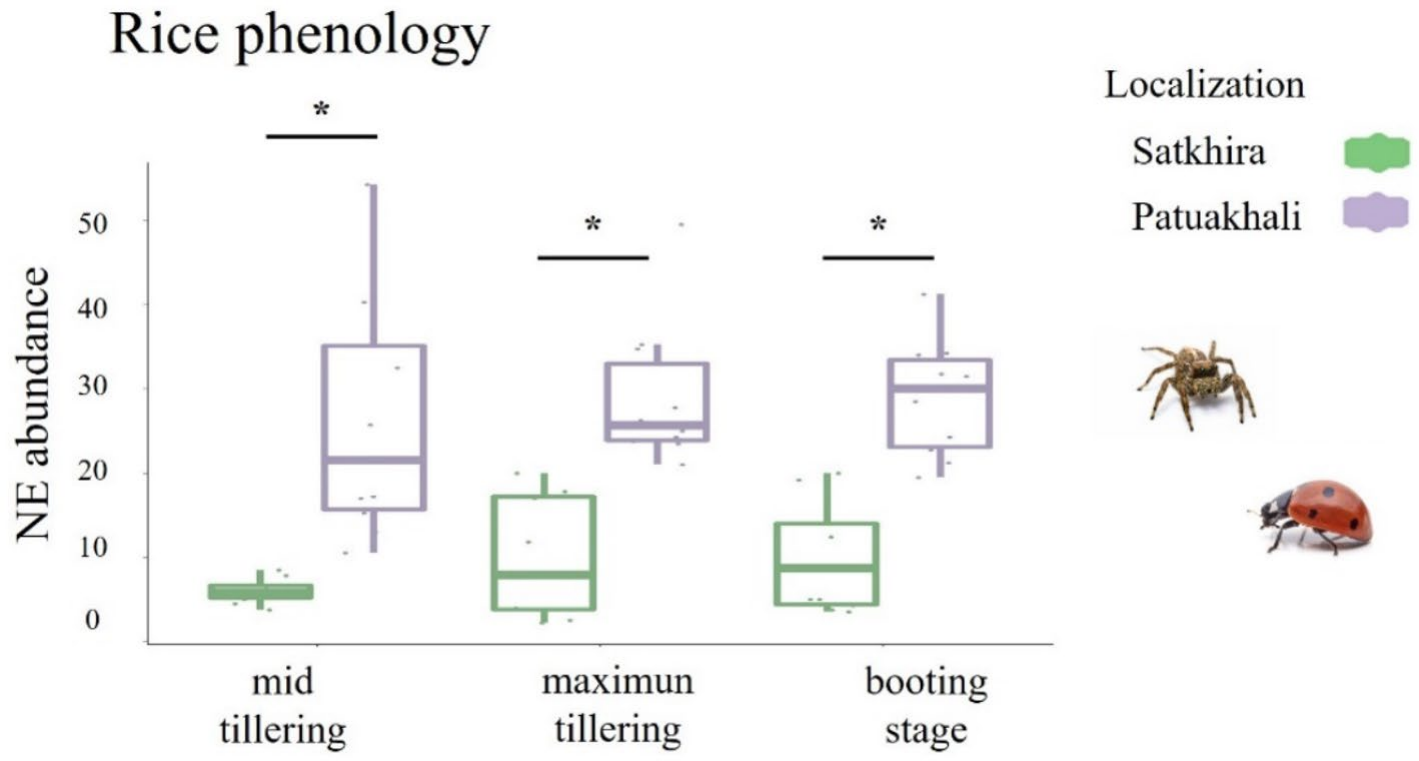
Effect of rice phenology on the abundance of natural enemies (NE) with the mean and standard deviation measured in Patuakhali and Satkhira regions. *Significant differences according to t-test (for Gaussian variables) (*p* < 0.05). Significant variation was observed between locations irrespective of rice phenology. Each boxplot represents the cumulative number of ladybird beetles and spiders.

Both region and plant phenology had an effect on natural enemy abundance (Fig. 3). We found significant differences in the abundance of natural enemies which were higher in Patuakhali (F_1,54_ = 91.57, *p* < 0.001) and when we compared phenology between regions, we found that abundance of natural enemies was higher in Patuakhali during mid tillering (t = 3.36, *df* = 1, *p* = 0.01), maximum tillering (t = 4.74, *df* = 1, *p* < 0.001) and booting stage (t = 4.73, *df* = 1, *p* < 0.001) (Fig. 3). No significant differences were found between rice phenology stages when compared inside each region (F_2,27_ = 0.73, *p* = 0.48).

**Figure 4:**
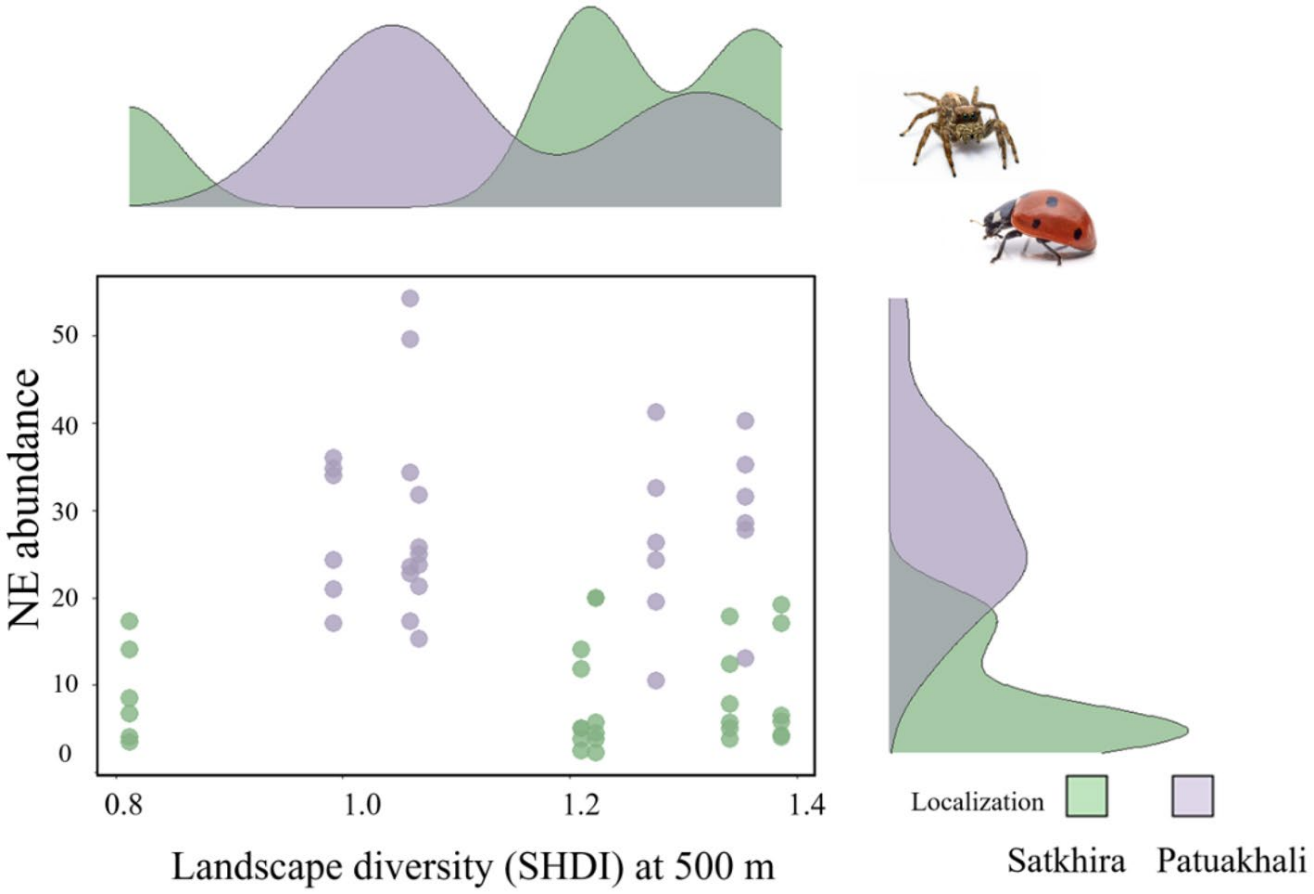
Density plot visualizes the distribution of natural enemy (NE) abundances across varying landscape diversity (SHDI) and regions.

We used density plots to show the distribution of the abundance of natural enemies in relation to the value of Shannon’s landscape diversity index (SHDI) in the two regions (Fig. 4). Fields in the Satkhira region had a range of low to high SHDI values and generally low natural enemy abundance, while fields in the Patuakhali region had overall higher SHDI values and greater abundance of natural enemies.

## Discussion

Managing agricultural landscapes to support ecosystem services such as natural pest suppression is a key aim of sustainable agriculture^13,30^. Increasingly, it is recognised that landscape structure including both composition and configuration can influence natural enemies and pest suppression^31,32^. However, very few studies have previously attempted to show the impact of landscape diversity on pest management in rice^14,33^. Here we show the effect of landscape diversity at multiple spatial scales on the two main natural enemies (ladybird beetles and spiders) of brown planthopper (BPH) and assess the amount of natural pest control services in rice field with implications for future landscape management as part of an Integrated Pest Management (IPM) approach.

Our study shows that naturally occurring levels of natural enemies provide significant pest suppression services to rice farmers. When natural enemies were excluded using cages, sentinel populations of BPH increased significantly in contrast to the uncaged controls, resulting in 15.3–37.0% higher grain yield loss. In contrast, BPH populations in the uncaged plots as well as the entire experimental fields remained low due to action of natural enemies. Several predators of BPH including ladybird beetles and spiders were found in all fields^34^ and were frequently observed on the mesh net attempting to enter the cages where BPH populations were increasing. The absence of natural enemies inside the cages allowed dense populations of BPH to develop, resulting in grain yield losses. This level of BPH infestation is common in some areas of Bangladesh^35^ where hot, humid, and moist conditions induce higher BPH infestations^36^. Our study shows that without application of chemical pesticide in crop field, naturally occurring levels of NE’s could provide significant pest suppression services to farmers. But our farmers are unaware of this natural pest suppression service and regularly use insecticide to control insect pests in rice, thus each field commonly receiving 2–4 insecticides applications per year in Bangladesh^37^. These practices have negative impact of natural enemies in rice field resulting planthopper outbreaks^29^. In addition, we calculated a biological control service index which shows significant negative impact on both pest density and grain yield loss. This service is one of ecological functions that depends on biodiversity and this effect is found due to naturally occurring predators on the population density of pests.

We found that natural enemies and pest abundances vary across the two rice production regions represent the areas of salinity-intrusion which can alter pest and natural enemy interactions^38^. The rice agroecosystem landscape in Satkhira is dominated by rice and forest patches and a significant proportion of urban areas, while in Patuakhali, rice fields were more often surrounded by fallow lands and waterbodies. These landscape features had significant effects on the abundance of natural enemies and BPH in rice fields. While the agricultural landscape structure in each region differed, overall, successful natural control of BPH was observed in both regions. Our results reveal that ladybird beetle abundance responds to landscape composition at multiple spatial scales, with the best model fit at the 500 m scale. However, spiders did not show landscape effects and they were most affected by rice phenology. Spiders and ladybird beetles can be affected by landscape variables higher than 1 km. The scales used in this study can partly explain the effect of the landscape variables on natural enemies, for most variables. Vegetation in rice bunds provides habitat for spiders which disperse into establishing rice crops^39^ however, the impacts of rice bunds and their functional connectedness on the community composition of rice arthropods, particularly at landscape sizes, are still poorly understood. We found that spiders are highly dependent on rice phenological stage. This finding may have different implications for cursorial spiders and web-building spiders, as they are affected differently by crop structure^40,41^. The latter may benefit from a more developed crop canopy at later phenological stages to build their webs.

In Satkhira, asynchronous rice cropping provides a mosaic of cultivated and fallow fields that provide predators and parasitoids with a constant supply of prey through time and space, potentially assisting them in avoiding spatial and temporal limitations^42^. Wilby et al.^43^ demonstrated how local heterogeneity (defined as crop stage) might affect rice-arthropod ecosystems at various phases of the rice plant. In contrast, synchronous rice cropping is common in Patuakhali could potentially promote more frequent use of chemical pesticides reducing natural enemy effectiveness. Despite this, higher abundances of natural enemies were detected in the fields located in Patuakhali during three stages of rice and BPH suppression was equivalent to that of Satkhira.

Efforts to improve pest control by managing habitats often focus on the connection between landscape structure, natural enemy abundance, and pest suppression^44^^,^^45,46^. Using a multi-scale method is particularly useful in such studies when the scale of the organism-landscape interaction is unknown^44^. Our results suggest that spiders, as generalist predators, are highly dependent on rice stages rather than landscape composition. In further studies both generalist and specialist groups of natural enemies should be compared to increase knowledge of their interactions in rice agroecosystems.

Much of the organization of the arthropod populations in sub-tropical rice agroecosystems in Bangladesh is explained by regional rather than fine-scale landscape variability (e.g., the impact of elevation as a proxy for climate and other landscape characteristics)^14,33^. We found significant regional impact on the abundance of ladybird beetles and spiders in each experimental plot (Fig. S2). These regional differences are mostly influenced by species mobility, and so differ between species^45^.

Local patterns of land use in the two regions also influenced natural enemy abundance in rice fields. We detected higher proportion of rice in the landscape around the experimental plots in fields located in Satkhira while, a significantly higher proportion of fallow land exists around the experimental fields at each scale explored in Patuakhali. To date only a few studies have examined the possible benefits of landscape heterogeneity or habitat alteration for the natural enemies of rice pests^,^^9,10,16–18^. In our study, the combination of a high proportion of fallow areas may allow spillover of natural enemies into rice crops. Specially, we use roads as an element in our analysis because they represent stable strips of perennial vegetation in the landscape. For example, mango, guava, banana, raintree, acacia, shrubs, and herbs are commonly cultivated in road edges in both regions (See supplementary photo 1). As such, roads are important elements of the landscape structure because provide multiple resources (food, alternative prey) or refuges for natural enemies in rice landscapes. These local landscapes around crop can play an important role in maintaining natural enemies and predation services that help regulate pest populations in crop field^46^. By providing resources like alternate prey or hosts, nectar and pollen for omnivores, appropriate microclimates, and habitat for various life stages, habitats with more complex vegetation often benefit natural enemies^11^. Thus, our result supports the enemy hypothesis which states that increasing vegetation complexity enhances natural enemy abundance and diversity as well as pest regulation^47^. Increasing plant diversity is frequently recommended as a critical management strategy to harness ecosystem services based on biodiversity since it is especially important to preserve numerous ecosystem services^46,48^. Although roads could also fragment elements in the landscape, a spillover effect from these old and mature natural habitats (relict patches of ancient natural habitat) to crop have been documented in other cropping systems^49^.

The abundance of ladybird beetles has also been shown to increase with the number of rice patches^14^. Smaller rice habitat patches combined with asynchronous rice fields and other crops could improve the success of mobile ladybeetles^40^. We show that areas close to roads are also likely to improve biological control in rice agroecosystems, more than small edges between rice patches. Moreover, fallow land reduces the application of chemical pesticides around rice fields, potentially reducing negative impacts on natural enemies favouring biocontrol services in experimental fields. Our farmers typically apply insecticides in rice fields without considering the pest status^37^. Overall, heterogeneity in the landscape composed of fallow lands seems to promote the abundance of ladybird beetles and the ecosystem service of natural pest control. Finally, the intensive use of insecticides in the Satkhira region may be decreasing overall natural enemy populations and thus the effectiveness of biological control.

We also found an effect of urbanization on predator species in neighbouring rice crops. Specifically, the proportion of urbanized land in the landscape had a negative effect on ladybird beetles that varied with scale. Urbanization mainly reduces the abundance of neighbouring rice fields and changes the phenology, physiology, and behaviour of herbivorous arthropods, as well as their community assemblage^50^. Landscapes are becoming increasingly urbanized, causing loss and fragmentation of natural habitats, with potentially negative effects on biodiversity^51^. Thus, urbanization may negatively affect the amount of natural colonization of rice by ladybird beetles coming from surrounding fields. This suggests that in further studies we need to include urban proportion as a potentially important variable which could play an important role in biological control.

The abundance of natural enemies is usually positively impacted by the diversity of the landscape^52^, though both positive and negative impacts have been observed in agroecosystems^31^. Interestingly, we found that although landscape diversity indexes were higher in the Satkhira region, even the lower level of landscape diversity in Patuakhali supported high natural enemy abundance, especially of the two main rice predators, ladybird beetles and spiders. Although natural non-crop habitats may not always have substantial pest control impacts, heterogeneous landscapes with a diversity of habitat types frequently support biodiversity and ecosystem services in agricultural systems^27,53^. Sometimes, landscape diversity effects could be masked by other factors such as the management of fields, or that other land uses represent a significant food and habitats than surrounding resources^54,55^, which in our case is reflected by the fallow lands within rice agroecosystem.

Resource availability can be disrupted by habitat fragmentation, and predators are typically more susceptible to fragmentation than their prey^56^. Our study shows that natural enemies benefit from agricultural landscapes comprised of small patches with increased edge density. Landscapes with high edge density increase the abundance of natural enemy and enhances pest control^32^. Rice arthropods’ capacity to migrate through the rice agroecosystem is presumably aided by the existence of edges^14^ and rice fields connected through an extensive network of bunds, generally with scarce semi-natural vegetation can perhaps provide alternate food resources or refugia to natural enemies^57,58^. Thus, the abundance of natural enemies is better explained by the presence of edges in the rice, rather than by connectivity of rice patches in the landscape.

Finally, several other factors could have influenced our results. For example, while cages prevent natural enemies from entering, they also limit pest dispersal and could artificially inflate pest abundance and damage. We also did not use insecticides in our experimental fields while surrounding rice fields typically received heavy insecticide use, particularly in Satkhira. Insecticides can have significant impact on the abundance of natural enemies in crop fields^59,60^ and many farmers in Bangladesh apply insecticides prophylactically rather than using an Integrated Pest Management (IPM) approach. Previous studies have shown that advising farmers not to use chemical insecticides during the early stage of rice production (up to 30–40 days after transplanting), enhances the effectiveness of biological control^8^. Thus, by not using insecticides in our experimental fields we favoured finding positive effects of pest suppression and prove the ability of naturally occurring natural enemies in pest regulation. This is usually recognized as ecosystem services as pest control service. However, the fact that natural enemies effectively controlled BPH throughout the experimental fields shows the resilience of this ecosystem service even in highly disturbed landscapes.

Pest outbreaks in rice regularly cause economic damage to rice growers, despite and in part because of their indiscriminate usage of insecticides. The results of the present study show that landscape composition can enhance the ecosystem service of natural pest control by conservation of naturally occurring predators. We show how multi-scale research findings could be utilized to promote ecologically sustainable landscape planning by identifying local management techniques that are appropriate for the landscape, specifically, the importance of wide (currently primarily road) edges and fallow lands in the surrounding local landscape. Specifically, these results suggest that promoting fallow lands and fragmented patches between growing rice fields along with decreasing the prophylactic use of insecticides could lead to more sustainable insect pest management in rice agroecosystem. Based on our results we recommend the following measures to support natural pest control as a key aim of a sustainable rice agriculture: (1) promote fallow lands and wide perennial edges between growing rice patches; (2) avoid the coincidence of large urban areas with rice agroecosystems and, (3) decrease the prophylactic use of insecticides in rice.

## Methods

### Study area

**Figure 5:**
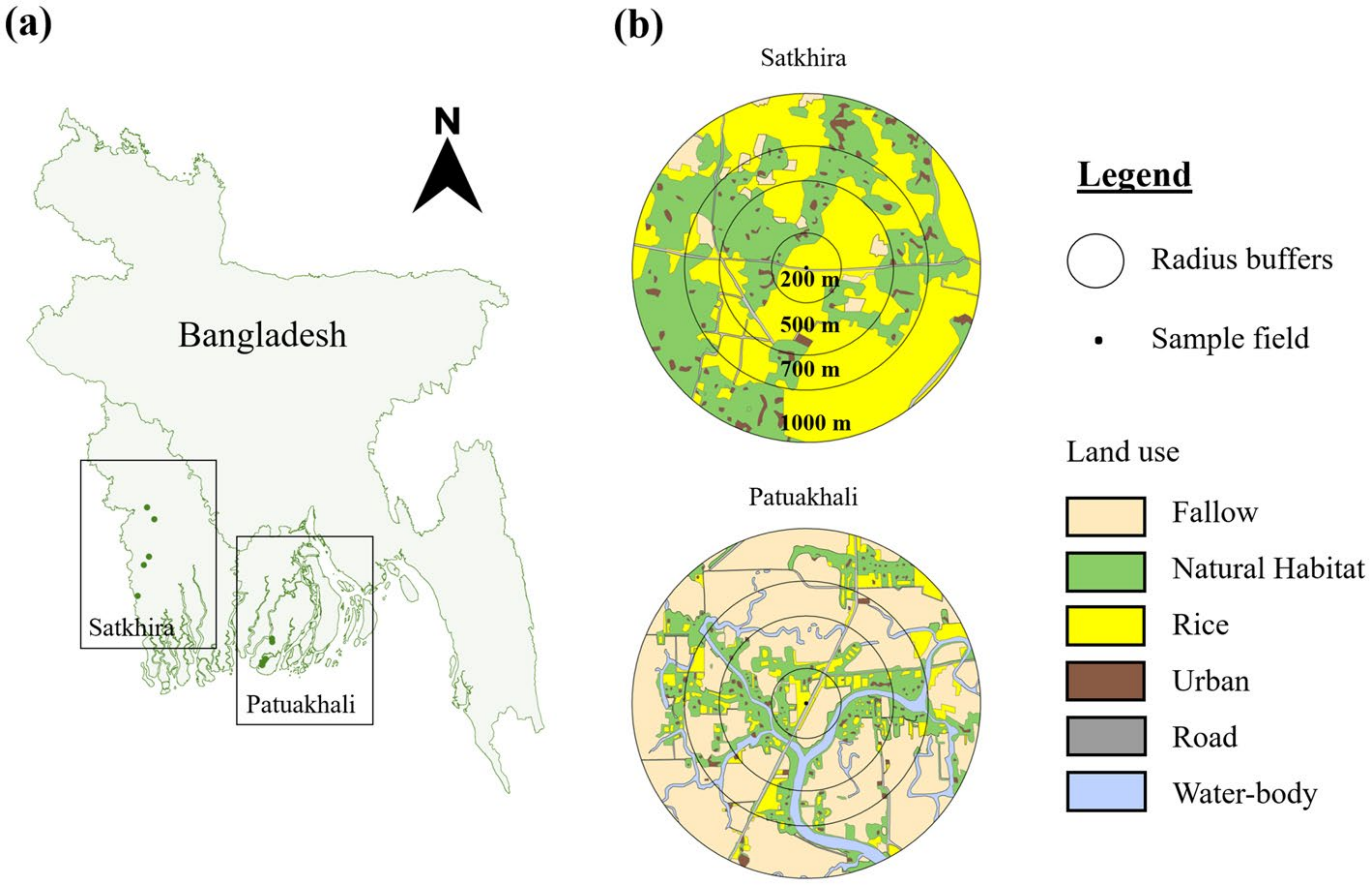
(**a**) Locations of the two study regions in Bangladesh (Patuakhali and Satkhira) and (**b**) examples of sampled rice field sites and mapping of land cover features within 200, 500, 700 and 1000 m radius buffers around the sampled fields. The map was generated using ArcGIS software 10.6.1 (Single permanent licence).

The study was conducted within the coastal belt of Bangladesh (Fig. 5). Two geographic regions were included: (i) Patuakhali, a coastal belt in south-eastern Bangladesh in Barishal division; and (ii) Satkhira, a traditional shrimp cultivation area in north-western Bangladesh in Khulna division (Fig. 5a). Both regions represent salinity-intrusion zones where rice production is increasingly challenged due to elevated soil salinity^44^. Within each region, 5 rice fields (sites) were selected, resulting in a total of 10 sites (Fig. 5b).

### Experimental fields

Experiments were conducted in two consecutive years during the Boro rice growing. The rice varieties used were BRRI dhan28 (2018–2019) and BRRI dhan29 (2019–2020) sourced from the Genetic Resource and Seed Division of Bangladesh Rice Research Institute (BRRI). Both varieties are similarly susceptible to the brown planthopper, *Nilaparvata lugens* (Stål) (Hemiptera: Delphacidae) the key pest of rice in these regions. Rice seedlings were raised in seedbed nurseries and transplanted in main experimental fields. Before transplanting rice seedlings, wet soil was prepared through ploughing followed by laddering according to typical farm procedures^36^. During the rice cultivation season, 40–45 d old seedlings were transplanted into the fields. The standard transplant spacing of 20 × 20 cm^2^ was followed. Urea, triple superphosphate (TSP), muriate of potash (MP), Zinc sulphate, and gypsum were used to supply N, P, K, Zn, and S at rates of 82, 15, 38, 10.6, and 2.7 kg/ha respectively. During final land preparations, the complete amount of TSP, MP, gypsum, and 1/3 of the urea was applied. The remaining urea was applied in two equal splits, 20 d after transplanting at the early tillering stage and 40 d after transplanting at the maximum tillering stage and timed to coincide with irrigation or naturally occurring wet soil conditions. No insecticides were used in the fields during the experiment.

### Assessment of pest control service

**Figure 6:**
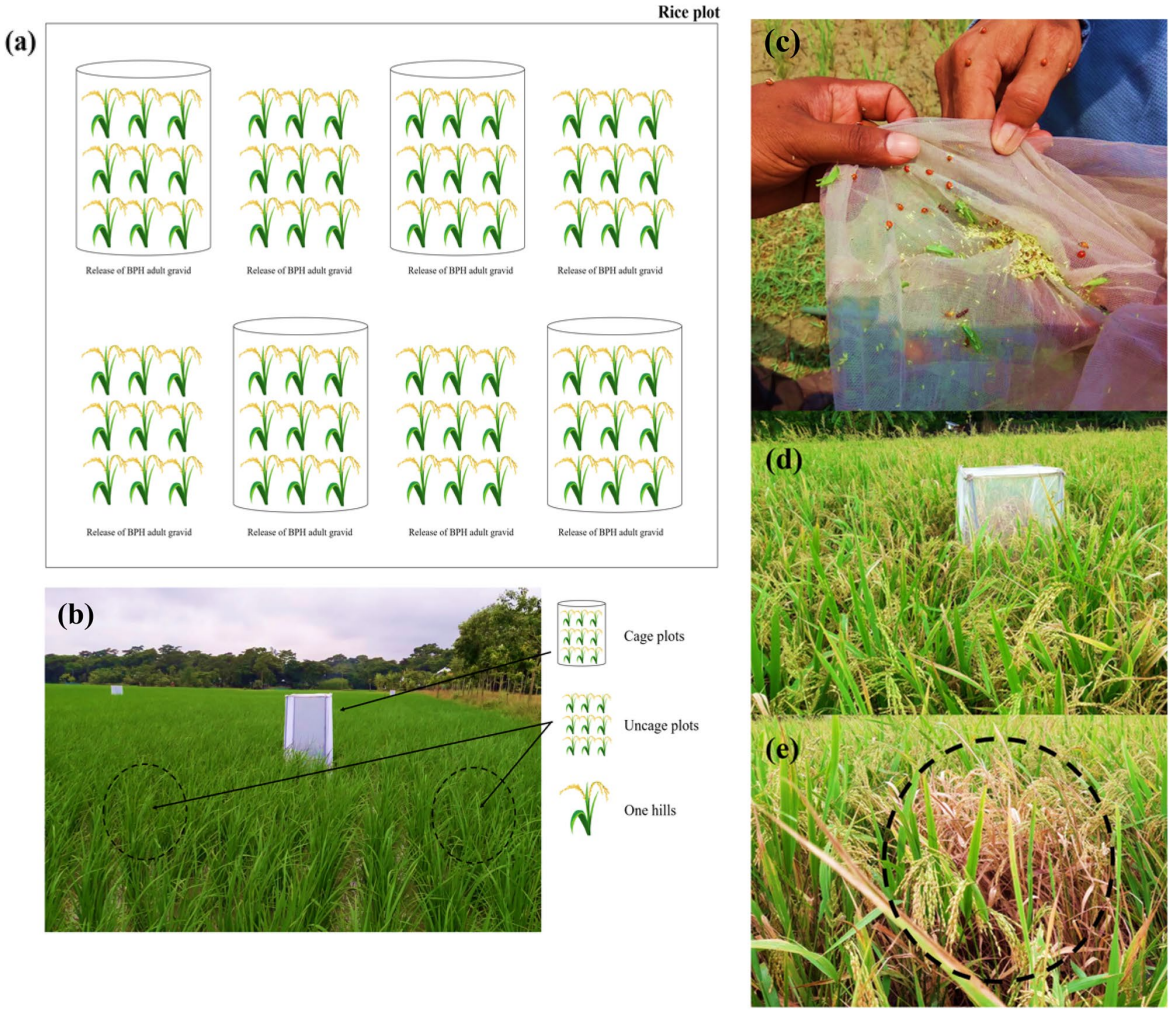
(**a**) Depiction of experimental design within each field, (**b**) photo of an example field site showing caged and uncaged plots, (**c**) sampling with a sweep net showing the abundance of ladybird beetles and other insects, (**d**) example of caged plot at rice maturity stage, (**e**) with caged removed to show extent of damage. The figure was prepared using MS PowerPoint software and pictures were taken by MP Ali and MMK Kabir from experimental fields.

To quantify pest control services, we released gravid female brown planthoppers (hereafter called BPH) into 8 plots within each rice field (Fig. 6). Each plot consisted of nine hills of rice and received 4 BPH in 2018–2019 and 10 in 2019–2020. One half of the plots (n = 4) were covered by mesh nylon nets (mesh width: 5.6 mm) that protected BPH from natural enemies (hereafter called caged plots, Fig. 6a,b), while the remaining 4 plots were left uncaged and exposed to natural enemies (hereafter called uncaged plots). Plots were infested on 01.03.2019 and 07.03.2020 and were monitored up to crop harvest. Brown planthoppers developing within each plot were counted by observing all plants and leaves on three occasions (7, 25 and 55 d post infestation). In addition, to assess the impact of pest suppression on crop yield, when rice reached maturity, we recorded rice yield components including grain/panicle, tillers/hill, and 1000-grain weight in all plots (Fig. 6d).

### Sampling of natural enemies

Arthropods were collected from experimental fields using a 40-cm diameter sweep net (mesh width: 5.6 mm) **(**Fig. 6b). Twenty complete sweeps were conducted at the canopy level of the plants with samples taken at random locations near the field centre between 09:00–11:00 h. Sampling occurred at three different stages of rice development (mid tillering, maximum tillering and booting stage). Arthropods not identified in the field were transferred to the lab and identified following Barrion and Litsinger^61^. Predators including ladybird beetles, spiders, rove beetles, damsel flies and green mirid bugs were observed. Considering that ladybird beetles and spiders are the main natural enemies of BPH, their abundance was used for further analysis. Our analyses were limited to data collected during the dry season (February to April) of each year as the composition of arthropod communities is known to vary between wet and dry seasons Heong et al.^62^.

### Mapping and landscape metrics

Land use data was downloaded from the MODIS 2018 MCD 12 Q1 V6 and Global land cover 2009 however the “agricultural” land use category did not specify crop type. To address this, we downloaded a set of 10 orthophotos corresponding to our experimental rice fields using SAS.Planet (http://www.sasgis.org/) with ESRI maps from the ArcGis Imagery gallery^63^.

During the dry season, the rice fields are fallow in the Barisal district. To confirm land use at the date of sampling, we used Google Earth (https://www.google.com/intl/es/earth/). Finally, using ArcGIS software 10.6.1^63^ we built a new shapefile with specific land uses. All landscape features were identified and mapped within a 200, 500, 700 and 1000 m of radius around each sampling site (Fig. 5b). Land cover composition was classified into the proportion of six categories: rice, fallow, natural habitat, waterbodies, urban and road. Using FRAGSTATS^64^ we quantified landscape diversity, Shannon’s Diversity Index (SHDI) and edge density, around sampling sites at each of the four spatial scales.

### Biological control services index

Based on the effect of natural enemies on BPH we created a biological control services index^65^ (BSI) for each study location and year as a measure of the level of pest suppression. We used the number of BPH counted in the cages 4 weeks after the initial release to calculate BSI as follows to reflect the proportional decline in the BPH population caused by natural enemies^66^:

$${\text{BSI}}_{{\text{i}}} = 1 - \frac{{N_{o,i} }}{{N_{c,i} }}$$

where No, i is the typical BPH count at site i in the open cages and Nc, i is the typical BPH count at site i in the closed cages.

### Statistical analyses

We first compared the sum of BPH in the two treatments (caged and uncaged) with region (Satkhira and Patuakhali) as a fixed factor, and sampling days as random factor using generalised linear mixed model (GLMM) using glmer with the negative binomial tendency. Secondly, a t-test for Gaussian data was used to analyse differences in grain hill yield in the two treatments with the interaction of region used to analyse differences of natural enemies abundances during three stage of rice phenology (mid tillering, maximum tillering and booting) in both regions.

The responses of natural enemies to rice landscape composition were analysed using generalised linear mixed model (GLMM) using glmer (for the negative binomial distribution) at each spatial scale. The response variables were the average abundance of ladybird beetles and spiders. Since each of two study areas has relatively distinct rice management practices, region and year nested structure of region/field were assigned as random effects. Phenology and, the percentage of fallow, urban areas, road, waterbodies, natural habitat in the landscape, edge density and SHDI index were included as fixed factors in all models. We employed multimodel inference using the four spatial scales which fits models with all feasible predictor combinations and weights them using the Akaike information criterion (AIC). This approach recognizes a “best” model according to data-based selection as well as the ranking and weighting of the remaining models in a pre-defined collection. For each candidate model, this technique generated AICc values and Akaike weights. The model average was calculated based of ΔAICc < 2 criteria. The choice of the optimal approximating model is an inference from the data that shows us which effects expressed by parameters our data can support. The fixed variables for each model were standardised (mean centred and scaled). The model residuals were graphically assessed using qq-plot and residual histograms to make sure that the criteria for normality and homoscedasticity were not violated. The best-fitting models for all spatial scales were those with the lowest AICc values. Finally, the relative significance of each predictor variable was shown with the best model (as a sum of Akaike weights of the best models in which each variable appeared). The statistical analyses were conducted using the *lme4*^67^ and the dredge function in the multimodel inference in the MuMIn package^68^, and ‘MASS’ in R version 3.6.2^69^.

### Ethical statement

The collection and use of any plant materials in this study is carried out in accordance with any National/International/Legislative/Institutional guidelines and regulations.

### Supplementary Information

Supplementary Information 1.

Supplementary Information 2.

## Data Availability

All data generated or analysed during this study are included in this published article and its supplementary information files.
