# Supplementary material for: Landscape structure influences natural pest suppression in a rice agroecosystem
Source: Sci Rep. 2023 Sep 21;13:15726. doi: 10.1038/s41598-023-41786-y (PMC10514064; doi:10.1038/s41598-023-41786-y)
Supplement: Supplementary file 1 — Supplementary Information 1. [file 41598_2023_41786_MOESM1_ESM.docx]

**Supplementary information**

**for**

**Landscape Structure Influences Natural Pest Suppression in a Rice Agroecosystem**

MP Ali^1*^, Gemma Clemente-Orta^2*^, MMM Kabir^1^, SS Haque^1^, M Biswas^3^, and Douglas A. Landis^4^


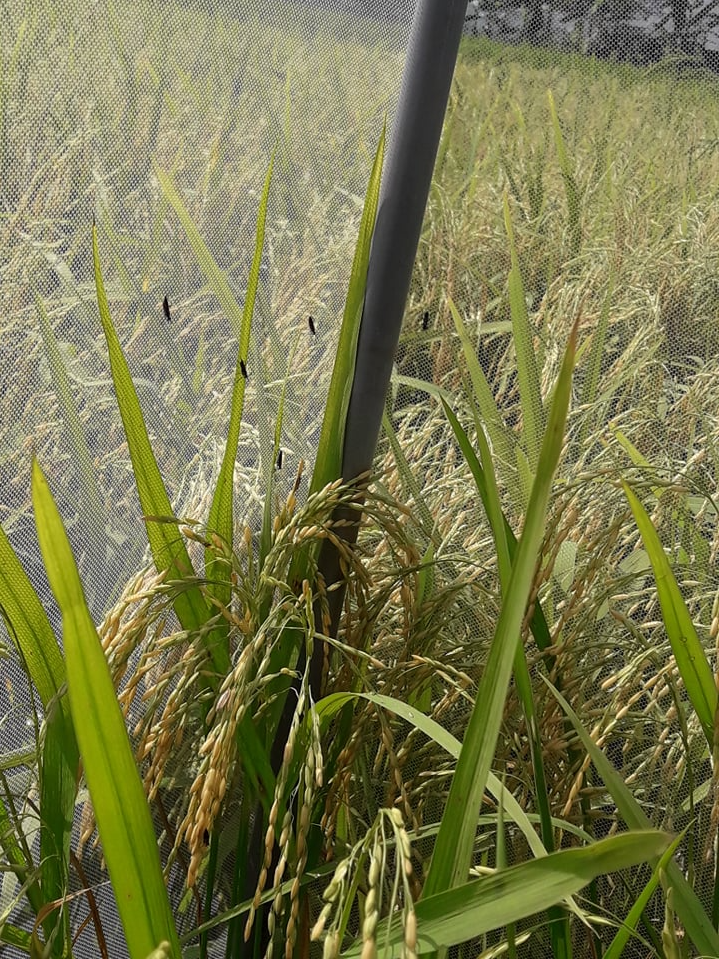


Figure S1: Natural enemies like rove beetles sat on the nylon mesh net of caged control plot. The picture was taken my MP Ali from experimental fields.


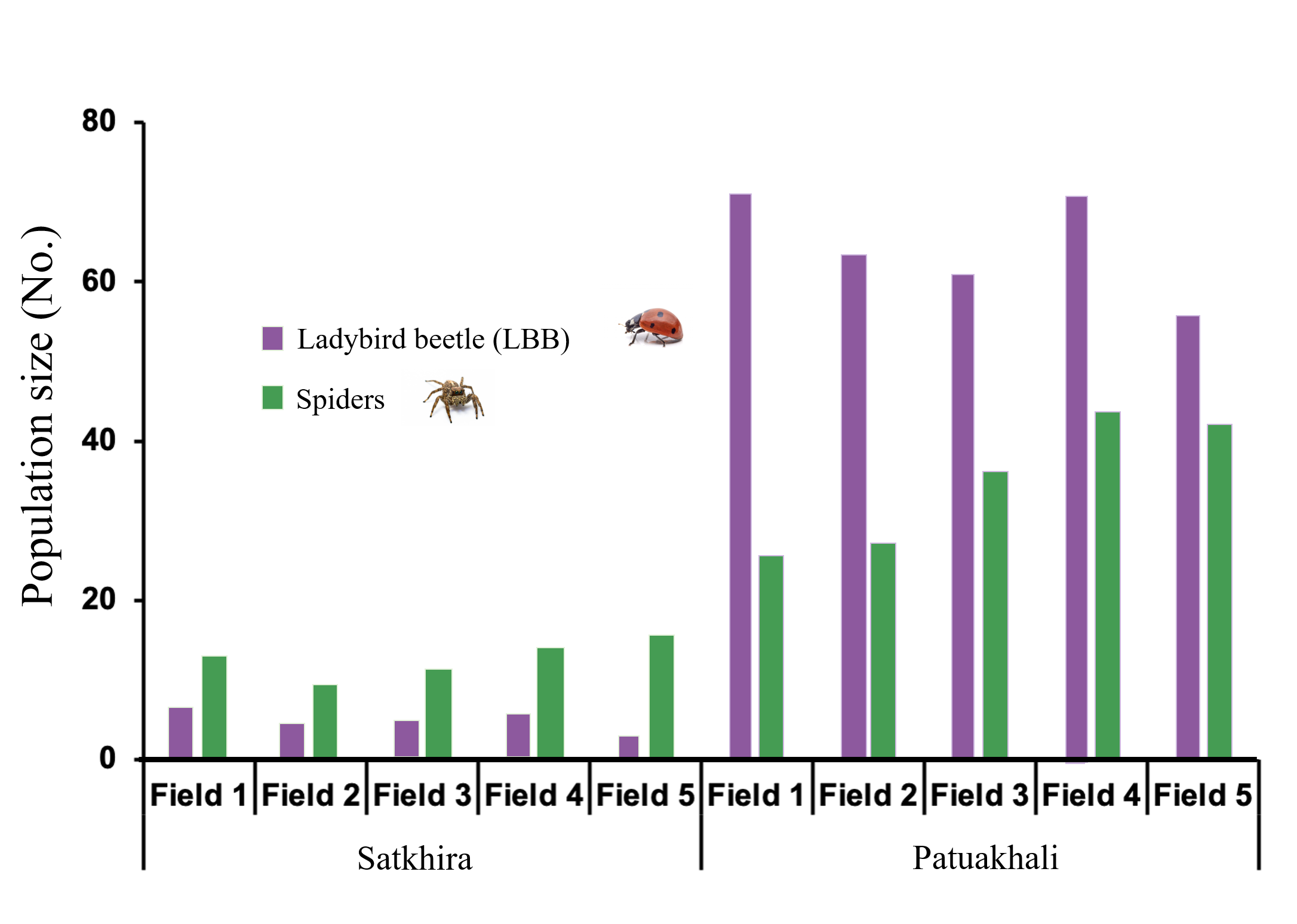


Figure S2: Abundance of lady bird beetle and spiders in experimental field at two geographical locations.


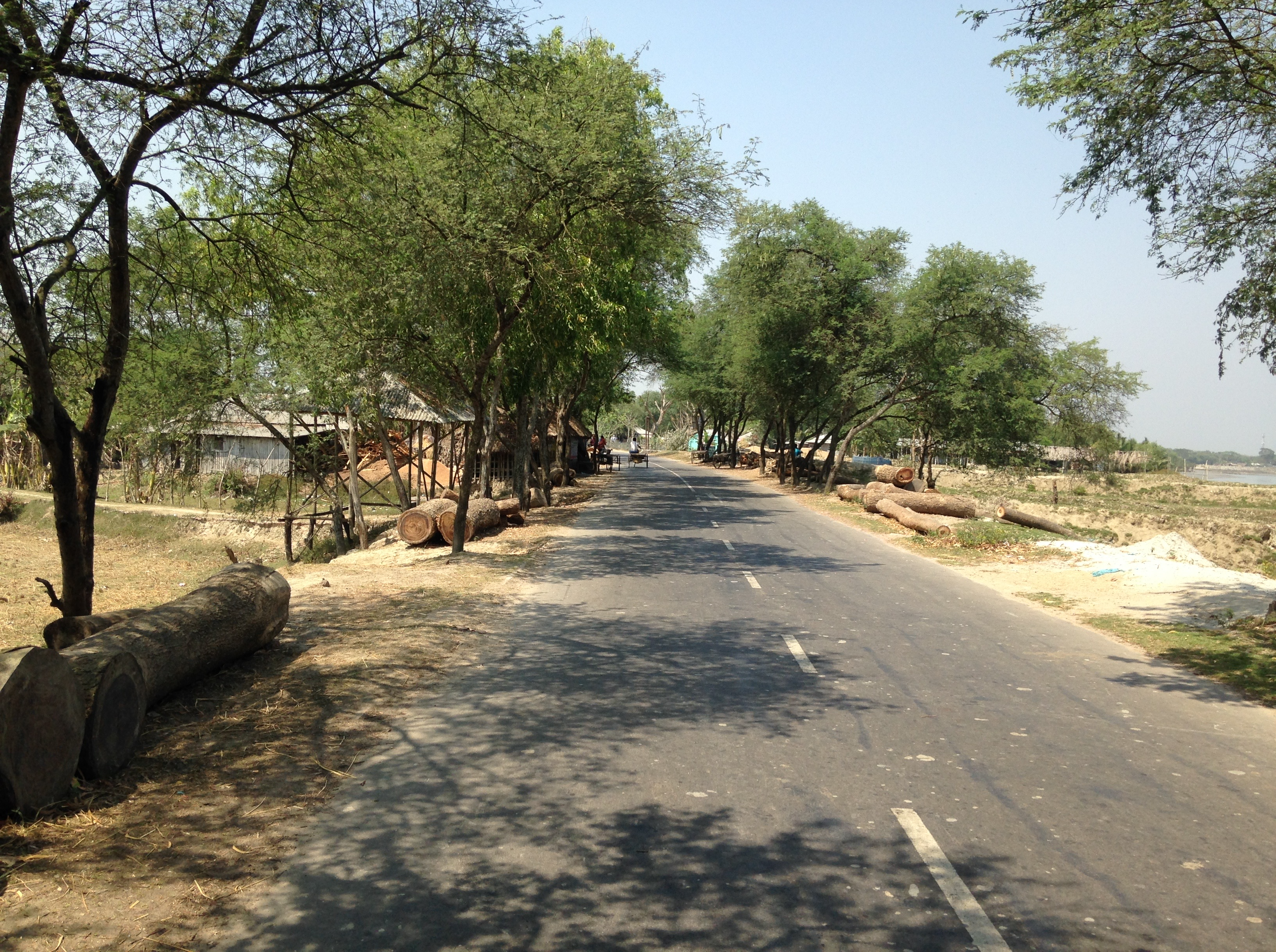


Photo S1: Picture of road near to experimental plots. The photo was taken by MMK Kabir from the experimental area.

Table S1. Most parsimonious model results of the best models explaining Lady bird beetle and spider abundance. All variables present in the best models (ΔAIC < 2) are presented; significant *p* values are in bold characters.

| **1000 m** |  |  |  | **700 m** |  |  |  | **500 m** |  |  |  | **200 m** |  |  |  | R2 |
| --- | --- | --- | --- | --- | --- | --- | --- | --- | --- | --- | --- | --- | --- | --- | --- | --- |
| **LBB** |  |  |  |  |  |  |  |  |  |  |  |  |  |  |  | 0.96 |
| **Variables** | **Est.** | **z** | ***p*** | **Variables** | **Est.** | **z** | ***p*** | **Variables** | **Est.** | **z** | ***p*** | **Variables** | **Est.** | **z** | ***p*** |  |
| (Intercept) | 2.18 | 2.11 | 0.03 | (Intercept) | 2.14 | 1.77 | 0.07 | (Intercept) | 1.75 | 2.19 | 0.03 | (Intercept) | 1.87 | 1.98 | 0.04 |  |
| **Water-bodies** | **0.2146** | **2.651** | **0.008111** | **Road** | **0.1446** | **2.053** | **0.0476** | **Fallow** | **0.383** | **2.467** | **0.0111** | Road | 0.07850 | 1.562 | 0.118247 | |
| Phenology-max | 0.24490 | 1.916 | 0.332291 | Fallow | 0.19496 | 1.380 | 0.167720 | **Road** | **0.122** | **2.071** | **0.0445** | **Urban** | **0.16** | **2.84** | **0.003321** |  |
| Phenology-mid | 0.06807 | 0.524 | 0.600048 | Phenology-max | 0.25184 | 1.931 | 0.053479 | **Urban** | **-0.191** | **2.165** | **0.0356** | NH | -0.09161 | 1.398 | 0.162043 | |
| Road | 0.06177 | 1.033 | 0.301427 | Phenology-mid | 0.06955 | 0.526 | 0.599177 | Phenology-max | 0.24538 | 1.925 | 0.054171 | Phenology-max | 0.24401 | 1.935 | 0.052984 | |
| Urban | 0.09066 | 0.887 | 0.374871 |  |  |  |  | Phenology-mid | 0.06562 | 0.507 | 0.612324 | Phenology-mid | 0.06596 | 0.515 | 0.606827 | |
|  |  |  |  |  |  |  |  |  |  |  |  | Fallow | 0.08808 | 1.036 | 0.300435 | |
|  |  |  |  |  |  |  |  |  |  |  |  |  |  |  |  |  |
|  |  |  |  |  |  |  |  |  |  |  |  |  |  |  |  |  |
| AICC | 324.6 |  |  | AICC | 327.62 |  |  | AICC | **324.9** |  |  | AICC | 325.411 |  |  |  |
| **Spiders** |  |  |  |  |  |  |  |  |  |  |  |  |  |  |  | 0.76 |
| **Variables** | **Est.** | **z** | ***p*** | **Variables** | **Est.** | **z** | ***p*** | **Variables** | **Est.** | **z** | ***p*** | **Variables** | **Est.** | **z** | ***p*** |  |
| (Intercept) | 2.36 | 7.68 | < 0.001 | (Intercept) | 2.36 | 7.68 | < 0.001 | (Intercept) | 2.35 | 7.61 | < 0.001 | (Intercept) | 2.35 | 7.9 | < 0.001 |  |
| Phenology-max | -0.11357 | 0.970 | 0.33229 | Phenology-max | -0.11310 | 0.971 | 0.33163 | Phenology-max | -0.11318 | 0.971 | 0.3318 | Phenology-max | -0.11464 | 0.987 | 0.32346 |  |
| **Phenology-mid** | **-0.2736** | **1.961** | **0.01123** | **Phenology-mid** | **-0.2763** | **1.951** | **0.04132** | **Phenology-mid** | **-0.2763** | **1.921** | **0.04111** | **Phenology-mid** | **-0.2763** | **1.951** | **0.04215** |  |
|  |  |  |  |  |  |  |  |  |  |  |  | NH | 0.08752 | 1.359 | 0.17429 |  |
|  |  |  |  |  |  |  |  |  |  |  |  | Fallow | -0.05587 | 0.852 | 0.39442 |  |
|  |  |  |  |  |  |  |  |  |  |  |  | Water-bodies | 0.02177 | 1.019 | 0.30825 |  |
|  |  |  |  |  |  |  |  |  |  |  |  |  |  |  |  |  |
|  |  |  |  |  |  |  |  |  |  |  |  |  |  |  |  |  |
| AICC | 361.933 |  |  | AICC | 361.492 |  |  | AICC | 361.536 |  |  | AICC | **360.081** |  |  |  |
